# Transfer of plant protection products from raspberry crops of Laszka and Seedling varieties to beehives

**DOI:** 10.1007/s10661-018-6491-z

**Published:** 2018-02-12

**Authors:** Bartosz Piechowicz, Karolina Mróz, Ewa Szpyrka, Aneta Zwolak, Przemysław Grodzicki

**Affiliations:** 1 Faculty of Biotechnology, Department of Analytical Chemistry, University of Rzeszów, Pigonia 1, 35-310 Rzeszów, Poland; 2 Laboratory for Research on Pesticide Residues, Institute of Plant Protection-National Research Institute, Langiewicza 28, 35-101 Rzeszów, Poland; 3 Faculty of Biology and Environmental Protection, Nicolaus Copernicus University, Lwowska 1, 87-100 Toruń, Poland

**Keywords:** Raspberries, Honeybees, Plant protection products, Pesticide residues, Consumption safety

## Abstract

Field studies were conducted to evaluate the transfer of active ingredients (AIs) of plant protection products (PPPs) to beehives. They were applied in two commodity red raspberry plantations of two varieties: Laszka (experiment 1) and Seedling (experiment 2). Samples of flowers, leaves, bees, brood, and honey were examined for the presence of chlorpyrifos, cypermethrin, difenoconazole, cyprodinil, and trifloxystrobin (experiment 1) and chlorpyrifos, boscalid, pyraclostrobin, cypermethrin, difenoconazole, and azoxystrobin (experiment 2). In experiment 1, the highest levels of trifloxystrobin were observed on the surface of flowers, (0.04 μg/flower) and for difenoconazole on the inside (0.023 μg/flower). Leaves contained only trace residues of cypermethrin and cyprodinil (0.001 μg/cm^2^ of leaves each) and trifloxystrobin (0.01 μg/cm^2^ of leaves) on the surface; inside the leaves, the highest levels of trifloxystrobin were observed (0.042 μg/cm^2^ of leaves). In experiment 2, boscalid was found on the surface and inside the flowers and leaves (0.063 and 0.018 μg/flower and 0.057 and 0.033 μg/cm^2^ of leaves, respectively). In bees, brood, and honey (experiment 1), chlorpyrifos was present in the highest quantity (7.3, 1.6, and 4.7 μg/kg, respectively). Additionally, cypermethrin and trifloxystrobin were found in bees, and trifloxystrobin was present in honey. Bees, brood, and honey from plantation 2 contained all studied AIs, with the highest levels of boscalid (28.6 μg/kg of bees, 37.0 μg/kg of brood, and 33.9 μg/kg of honey, respectively). In no case did the PPP residues in honey exceed acceptable maximum residue levels (MRLs)—from a formal and legal point of view, in terms of the used plant protection products, the analysed honey was fit for human consumption.

## Introduction

Pollinator insects’ work is currently valued at € 153 billion a year, i.e. 9.5% of global agricultural production (Gallai et al. 2009). In Brazil alone, the value of their work performed is estimated at nearly $ 12 billion (Giannini et al. 2015). On a global scale, the honeybee is the most important pollinator of all cultivated plants. Sanjerehei (2014) estimates the participation of bees in the pollination process at 86.8% of all the work of pollinators, with a profit that is 54-fold higher than the value of honey alone. The calculated total value of work performed by bees is increasingly used to estimate the profit generated by pollinating insects and the losses caused by a shortage of bees in some areas. Majewski (2014) showed that a decrease in the number of honeybee colonies in Poland caused a decline in total crops valued at approximately € 728.5 million a year. Honeybees are a species of crucial importance for growth and development of the majority of chemically protected crop plants (Delaplane and Mayer 2000), but they are sensitive to the residues of active ingredients (AIs) of plant protection products (PPPs) (Thompson and Wilkins 2003; Chauzat et al. 2009; Glavan and Božič 2013; Williamson and Wright 2013).

The raspberry requires careful, intense protection against diseases and pests (Sadło et al. 2014; Sadło et al. 2015; Sadło et al. 2018). In 2015, in Poland, 79.895 t of raspberries were harvested from a total area of 27.375 ha (GUS 2016a). The red raspberry (*Rubus idaeus* L.) is a species of perennial plant from the Rosaceae family (*Rosaceae* Juss.), with a height reaching 2 m. In Poland, three varieties are grown: wild raspberry, summer-cultivated raspberry, and autumn-cultivated raspberry. Among them, summer raspberries are provided the most intense protection, as they are at risk of infections caused by numerous pathogens (including *Gleosporium venetum*, *Agrobacterium tumefaciens*, *Phragmidium rubi*-*idaei*, *Botrytis cinerea*, *Didymella applanata*, *Leptosphaeria coniothyrium*, *Fusarium* sp., and *Phytophthora fragariae*) and infestation with pests (e.g. *Incurvaria rubiella*, *Aphis* sp., *Cacoecia rosana*, *Resseliella theobaldi*, *Anthonomus rubi*, *Byturus tomentosus*, *Phyllocoptes gracilis*, and *Melolontha melolontha*) (Rusnak 2011). Therefore, raspberry plantations require careful protection during their flowering, which coincides with the fruiting period.

Despite a risk associated with the use of PPPs, and fungicides in particular, beekeepers frequently install hives in the vicinity of raspberry plantations. Its flowers are very intensely visited by bees, and the honey yield from 1 ha of a plantation can reach 150 kg. Bushes themselves are very reliable in terms of nectar production, even under less favourable weather conditions (Ministry of Agriculture 2017). Their flower anthers are easily accessible for bees, as indicated by Colwell et al. (2017); this may explain their increased visits from such insects. Fertilising of crops likely leads to pollen that is richer in amino acids than that of wild plants (Atasay et al. 2013).

Numerous studies have demonstrated that worker honeybees (*Apis mellifera*) collecting pollen and nectar from entomophilous plants can also carry various contaminants to a hive (Chauzat et al. 2009; Cresswell and Thompson 2012; Oruc et al. 2012). Some pesticides used for the protection of raspberry plantations against pests and diseases accumulate in their bodies and pollute honeybee products (Rissato et al. 2006). Therefore, some reports indicate that transfer of some AIs of PPPs to beehives is possible (Panseri et al. 2014; Piechowicz et al. 2018a, b, c).

Our study was aimed to determine whether, and to what extent, the AIs of PPPs might be carried from raspberry plants to beehives located in the immediate vicinity of crops.

## Materials and methods

### Field experiments

Two field experiments were performed. Experiment 1 was conducted from May 27 to June 23 on a red raspberry plantation (Laszka variety) located in Grabówka-Kolonia (Lublin Province, Poland), and experiment 2 was conducted from May 27 to July 2, at a red raspberry plantation (Seedling variety) located approximately 4 km from the plantation used in experiment 1.

These plantations were protected using conventional methods, in accordance with current programs. All preparations were applied according to the posted labels. The sprayer was a model RA 10/80 (Lochmann, Vilpiano, Italy) with ALBUZ ATR 80 nozzles. All treatments with insecticides were performed in the evening, at the end of circadian period of honeybee foraging. Treatments with fungicides were performed at any time of the day, without preserving the prevention period for bees.

Four honeybee colonies were used in each experiment (experiments 1 and 2). They were transported from the area where bees had no contact with pesticides and were placed between bushes on the raspberry plantations of an area of 0.8 ha (Laszka) and 0.7 ha (Seedling) on May 17, 2015.

On each raspberry plantation (experiments 1 and 2), four plant rows were selected for the study, each approximately 150 m long. One laboratory sample of leaves was collected from randomly selected plants in each row, from which an analytical portion consisting of 16 disks of 1 cm in diameter was cut. From the same randomly selected plants in each of the four selected rows, one analytical portion of eight flowers was collected. For the experiment, only fully developed leaves and flowers were collected from the external part of plants most exposed to the used preparation and environmental conditions. Every week during the field trial, laboratory samples of honeybee workers (workers retrieved from frames), brood (the cut pieces of honeycombs, from which the brood was extracted from freshly sealed cells), and honey (collected from non-sealed cells) were collected from each of the four hives.

#### Spraying programme at the raspberry variety Laszka plantation (experiment 1)

Eleven formulations, including six insecticides, four fungicides, and one herbicide, were used on the red raspberry plantation (Laszka variety). A detailed programme for the plantation protection against diseases and pests in 2015 is shown in Table 1. Because it was not possible to detect all tested active substances with the microcell electron capture detector (μECD) and the nitrogen-phosphorus detector (NPD) detector, in the collected samples, only residues of chlorpyrifos (soil application), cypermethrin, difenoconazole, cyprodinil, and trifloxystrobin (foliar application) were analysed.

**Table 1:** Protection programme for red raspberry (Laszka variety)

Application date	PPP, trade name	AI, common name	Chemical group	Application rate of PPP (L/ha or kg/ha)	Application rate of AI (kg/ha)
April 7	Dursban 480 EC (I^d^)*	Chlorpyrifos	Organophosphate	1.0	0.480
April 10	Bi 58 Nowy 400 EC (I^s^)	Dimethoate**	Organophosphate	1.0	0.580
April 27	Vertigo 018 EC (I^c,d^)	Abamectin**	Macrocyclic lactone	0.5	0.009
May 5	Basta 150 SL (H^d^)	Glufosinate-ammonium**	Amino-phosphonate	3.0	0.450
May 14	Score 250 EC (F^s^)	Difenoconazole	Triazole	0.4	0.100
May 15	Dursban 480 EC (I^d^)	Chlorpyrifos	Organophosphate	4.0	1.920
June 2	Zato 50 WG (F^s^)	Trifloxystrobin	Strobilurin	0.2	0.100
June 3	Cyperkill Super 25 EC (I^c^)	Cypermethrin	Pyrethroid	0.125	0.016
June 6	Calypso 480 SC (I^c^)	Thiacloprid**	Neonicotinoide	0.2	0.096
June 11	Switch 62,5 WG (F^c,d^)	Cyprodinil	Anilinopyrimidine	1.0	0.375
June 11	Pomarsol Forte 80 WG (F^c^)	Thiram**	Dithiocarbamate	2.0	1.600
June 16	Mospilan 20 SP (I^s^)	Acetamiprid**	Neonicotinoide	0.3	0.060

*I* insecticide, *H* herbicide, *F* fungicide

*applied to soil through an irrigation system in 10,000 L/ha, **AIs not determined

^s^systemic mode of action

^c^contact mode of action

^d^deep-seated mode of action

#### Spraying programme of the raspberry Seedling variety plantation (experiment 2)

Nine PPPs, including four insecticides and five fungicides, were used on the plantation of the red raspberry (Seedling variety). A detailed programme for the protection of the plantation of that variety in 2015 is shown in Table 2. It was not possible to detect all used AIs with the μECD and NPD detectors. In the collected samples, only residues of chlorpyrifos (soil application) and of boscalid, pyraclostrobin, cypermethrin, difenoconazole, and azoxystrobin (foliar application) were analysed.

**Table 2:** Protection programme of raspberry plantation (Seedling variety)

Application date	PPP, trade name	AI, common name	Chemical group	Application rate of PPP (L, kg/ha)	Application rate of AI (kg/ha)
March 23	Treol 770 EC (I^c^)	Paraffin oil**	Hydrocarbons	20	–
May 7	Amistar 250 SC (F^s^)*	Azoxystrobin	Strobilurin	0.5	0.125
May 9	Score 250 SC (F^s^)	Difenoconazole	Triazole	0.4	0.080
May 14	Mythos 300 SC (F^c,d^)	Pyrimethanil**	Anilinopyrimidine	3.0	0.900
May 14	Dursban 480 EC (I^d^)*	Chlorpyrifos	Organophosphate	4.0	1.92
May 16	Mospilan 20 SP (I^s^)	Acetamiprid**	Neonicotinoide	0.2	0.040
May 29	Cyperkil Super 250 EC (I^c^)	Cypermethrin	Pyrethroid	0.15	0.0375
June 23	Bellis 38 WG (F^s^)	Boscalid pyraclostrobin	Anilide Strobilurin	1.0	0.252 0.0192
June 30	Signum 33 WG (F^s^)	Boscalid pyraclostrobin	Anilide Strobilurin	1.8	0.4806 0.1206

*I* insecticide, *H* herbicide, *F* fungicide

*applied to soil through an irrigation system in 10,000 L/ha, **AIs not determined

^s^systemic mode of action

^c^contact mode of action

^d^deep-seated mode of action

### Extraction for pesticide residue determination in leaves and flowers

#### Surface pesticide residues

Analytical portions of raspberry flowers (eight flowers) or leaves (16 disks of 1 cm in diameter) were placed in bottles with petroleum ether (ca. 30 mL, Chempur, Poland). After transport to the laboratory, the bottle contents were intensively shaken for about 0.5 min and then filtered through anhydrous sodium sulphate (VI) into a 50-mL calibrated flask to remove traces of moisture. The analytical portions were rinsed three times with 5 mL of petroleum ether, and washings were used to rinse the filtration paper and sulphate.

#### Incurred pesticide residues

After extraction of surface residues with petroleum ether, four analytical portions of flowers collected on a given day were combined into single samples to increase the residue concentration in final extract. Likewise, leaf samples were processed. They were homogenised in the Waring Commercial 8010 EG blender (Waring, USA) with 100 mL of distilled water and 150 mL of acetone (Chempur, Poland) and filtered on the Büchner’s funnel under vacuum. The blender jar was flushed with 50 mL of acetone, and the washings were used to rinse the filter cake. One-fifth of the obtained filtrate volume was used for further analyses. The filtrate was transferred to a separatory funnel together with 100 mL of 2.5% sodium sulphate (VI) (Chempur, Poland) solution. Pesticide residues were extracted three times with 20, 10, and 10 mL of dichloromethane (Chempur, Poland). The combined extracts were evaporated to dryness, dissolved in approximately 10 mL of petroleum ether, and purified on a Florisil (Chempur, Poland) mini-column (Sadło et al. 2014; Sadło et al. 2015). Pesticides were eluted with 70 mL of a 3:7 (*v*/*v*) ethyl ether-petroleum ether (Chempur, Poland) mixture, and then with 70 mL of a 3:7 (*v*/*v*) acetone-petroleum ether mixture. The solvents were evaporated to dryness, and the residues were transferred quantitatively with petroleum ether to a 10-mL measuring flask.

#### Extraction for pesticide residue determination in honey, honey bee workers, and brood

An analytical portion of 5 g of animals or honey was shaken with 5 mL of distilled water and 10 mL of acetonitrile (Chempur, Poland). Then, a mixture of salts containing 4 g of anhydrous magnesium sulphate (VI) (Chempur, Poland), 1 g of sodium chloride (Chempur, Poland), 1 g of trisodium citrate (Chempur, Poland), and 0.5 g of sesquihydrate disodium hydrogen citrate (Chempur, Poland) was added. The contents were shaken for 2 min and centrifuged for 5 min at 4500 rpm at 21 °C. Six millilitres of the acetonitrile phase of the obtained extract was transferred to a polypropylene test tube containing 150 mg of PSA (primary secondary amine) (Agilent, USA) and 900 mg of anhydrous sodium sulphate (VI) (Chempur, Poland). The extract was vigorously shaken for 2 min and centrifuged for 5 min under conditions as above. Four millilitres of the extract was collected and transferred to a glass tube, evaporated to dryness on a rotary evaporator Heidolph Laborota 4000 Efficient (Heidolph, Germany), and dissolved in 4 mL of petroleum ether (Chempur, Poland).

#### Chromatographic determination of pesticide residues

The obtained extracts were analysed using an Agilent 7890 (Agilent, USA) gas chromatograph equipped with the μECD and NPD detectors. The chromatograph was controlled using ChemStation software and equipped with the autosampler and an HP-5MS column (30 m × 0.32 mm × 0.25 μm). The following conditions were used during the instrumental analysis: NPD detector temperature—300 °C, μECD detector—260 °C, and injector temperature—250 °C. The oven temperature was programmed as follows: 100 °C—0 min → 10 °C/min → 180 °C—4 min → 3 °C/min → 220 °C—15 min → 10 °C/min → 260 °C—11 min; the total analysis time was 55.3 min. The injection volume was 1 μl.

## Analytical standards

Certified pesticide analytical standards were obtained from Ehrenstorfer (Germany) and from the Institute of Industrial Organic Chemistry (Poland). For linearity determinations, standard solutions in a petroleum ether were prepared using the following concentrations of the standard: 0.002, 0.01, 0.05, 0.10, 0.50, and 1.0 mg/kg. Linearity was described with determination coefficients (*R*^2^ > 0.99). Excellent linearity was achieved for the studied pesticides when using matrix-matched standards. The lowest limits of quantifications (LOQs) were 0.001 μg per single flower or cm^2^ of leaf, and 0.1 μg per kg of honeybee, brood and honey.

## Data analysis

The residue contents (*R*_i_) were expressed either as micrograms per single flower, or as micrograms per square centimetre of leaves, or as micrograms per kilogram of honeybee workers, brood, and honey. The mean values (*R*_M_) and total residue levels for all substances (Tables 3 and 4) found in four samples collected on each sampling date were calculated by dividing the total obtained value of pesticide residues (*R*_i_) by the number of samples, *n* = 4.

**Table 3:** Mean residues (± standard deviations) of pesticides in flowers and leaves of red raspberry (Laszka variety) and in bees, brood, and honey

Sampling date	Chlorpyrifos	Cypermethrin	Difenoconazole	Cyprodinil	Trifloxystrobin
Residues on flower surface (μg/single flower)					
May 27	< LOQ	< LOQ*	< LOQ	< LOQ	< LOQ
June 3	< LOQ	< LOQ	< LOQ	< LOQ	0.040 ± 0.010
June 10	< LOQ	0.003 ± 0.000	< LOQ	< LOQ	0.011 ± 0.007
June 17	< LOQ	< LOQ	< LOQ	< LOQ	< LOQ
June 23	< LOQ	< LOQ	< LOQ	< LOQ	< LOQ
Residues in flowers (μg/cm^2^)					
May 27	0.009	< LOQ	0.023	< LOQ	< LOQ
June 3	0.012	< LOQ	0.023	< LOQ	< LOQ
June 10	0.003	< LOQ	< LOQ	< LOQ	< LOQ
June 17	< LOQ	< LOQ	< LOQ	0.022	< LOQ
June 23	< LOQ	< LOQ	< LOQ	0.007	< LOQ
Residues on leaf surface (μg/cm^2^)					
May 27	< LOQ	< LOQ	< LOQ	< LOQ	< LOQ
June 3	< LOQ	< LOQ	< LOQ	< LOQ	0.010 ± 0.008
June 10	< LOQ	0.001 ± 0.001	< LOQ	< LOQ	< LOQ
June 17	< LOQ	0.001 ± 0.000	< LOQ	< LOQ	< LOQ
June 23	< LOQ	< LOQ	< LOQ	0.001 ± 0.001	< LOQ
Residues in leaves (μg/cm^2^)					
May 27	0.004	< LOQ	< LOQ	< LOQ	< LOQ
June 3	0.004	< LOQ	< LOQ	< LOQ	0.042
June 10	< LOQ	< LOQ	< LOQ	< LOQ	0.024
June 17	< LOQ	< LOQ	< LOQ	0.016	0.011
June 23	< LOQ	< LOQ	< LOQ	0.033	0.002
Residues in bees (μg/kg)					
May 27	< LOQ	< LOQ	< LOQ	< LOQ	< LOQ
June 3	< LOQ	< LOQ	< LOQ	< LOQ	4.8 ± 3.7
June 10	2.7 ± 1.2	2.3 ± 2.7	< LOQ	< LOQ	< LOQ
June 17	7.3 ± 5.7	< LOQ	< LOQ	< LOQ	1.3 ± 2.6
June 23	< LOQ	< LOQ	< LOQ	< LOQ	< LOQ
Residues in brood (μg/kg)					
May 27	< LOQ	< LOQ	< LOQ	< LOQ	< LOQ
June 3	< LOQ	< LOQ	< LOQ	< LOQ	< LOQ
June 10	0.4 ± 0.4	< LOQ	< LOQ	< LOQ	< LOQ
June 17	1.6 ± 1.1	< LOQ	< LOQ	< LOQ	< LOQ
June 23	< LOQ	< LOQ	< LOQ	< LOQ	< LOQ
Residues in honey (μg/kg)					
May 27	< LOQ	< LOQ	< LOQ	< LOQ	< LOQ
June 3	< LOQ	< LOQ	< LOQ	< LOQ	< LOQ
June 10	3.5 ± 1.2	< LOQ	< LOQ	< LOQ	< LOQ
June 17	4.7 ± 2.2	< LOQ	< LOQ	< LOQ	3.1 ± 2.6
June 23	2.1 ± 1.5	< LOQ	< LOQ	< LOQ	4.1 ± 3.3

**Table 4:** Mean residues (± standard deviation) of used pesticides in flowers and leaves of red raspberry (Seedling variety) and in bees, brood and honey

Sampling date	Chlorpyrifos	Boscalid	Pyraclo-strobin	Cyper-methrin	Difeno-conazole	Azoxystrobin
Residues on flower surface (μg/single flower)						
May 27	< LOQ	< LOQ	< LOQ	< LOQ	0.002 ± 0.000	< LOQ
June 3	< LOQ	< LOQ	< LOQ	0.028 ± 0.005	< LOQ	< LOQ
June 10	0.002 ± 0.001	< LOQ	< LOQ	0.013 ± 0.007	< LOQ	< LOQ
June 17	< LOQ	< LOQ	< LOQ	0.008 ± 0.002	< LOQ	< LOQ
June 23	< LOQ	< LOQ	< LOQ	0.002 ± 0.002	< LOQ	< LOQ
July 2	< LOQ	0.063 ± 0.011	0.013 ± 0.005	< LOQ	< LOQ	< LOQ
Residues in flowers (μg/cm^2^)						
May 27	0.013	< LOQ	< LOQ	< LOQ	0.018	0.010
June 3	0.004	< LOQ	< LOQ	< LOQ	0.013	0.008
June 10	0.005	< LOQ	< LOQ	< LOQ	< LOQ	< LOQ
June 17	0.002	< LOQ	< LOQ	< LOQ	< LOQ	< LOQ
June 23	0.005	< LOQ	< LOQ	< LOQ	< LOQ	< LOQ
July 2	0.002	0.018	0.005	< LOQ	< LOQ	< LOQ
Residues on leaf surface (μg/cm^2^)						
May 27	< LOQ	< LOQ	< LOQ	< LOQ	0.003 ± 0.001	< LOQ
June 3	< LOQ	< LOQ	< LOQ	0.055 ± 0.007	< LOQ	< LOQ
June 10	< LOQ	< LOQ	< LOQ	0.034 ± 0.010	< LOQ	< LOQ
June 17	< LOQ	< LOQ	< LOQ	0.021 ± 0.018	< LOQ	< LOQ
June 23	< LOQ	< LOQ	< LOQ	0.022 ± 0.009	< LOQ	< LOQ
July 2	< LOQ	0.057 ± 0.34	0.025 ± 0.011	0.017 ± 0.016	< LOQ	< LOQ
Residues in leaves (μg/cm^2^)						
May 27	0.006	< LOQ	< LOQ	< LOQ	0.006	0.003
June 3	0.005	< LOQ	< LOQ	< LOQ	0.003	< LOQ
June 10	0.002	< LOQ	< LOQ	< LOQ	< LOQ	< LOQ
June 17	0.002	< LOQ	< LOQ	< LOQ	< LOQ	< LOQ
June 23	0.002	< LOQ	< LOQ	< LOQ	< LOQ	< LOQ
July 2	< LOQ	0.033	0.019	< LOQ	< LOQ	< LOQ
Residues in bees (μg/kg)						
May 27	3.9 ± 1.6	< LOQ	< LOQ	< LOQ	1.6 ± 6.1	1.0 ± 1.2
June 3	3.7 ± 1.4	< LOQ	< LOQ	22.9 ± 17.5	12.2 ± 10.4	6.5 ± 5.1
June 10	2.9 ± 1.7	< LOQ	< LOQ	14.8 ± 8.0	5.9 ± 10.3	0.4 ± 0.4
June 17	1.8 ± 0.4	< LOQ	< LOQ	6.0 ± 5.0	2.9 ± 2.9	0.2 ± 0.3
June 23	1.6 ± 0.6	< LOQ	< LOQ	2.9 ± 2.9	7.2 ± 7.0	0.5 ± 0.8
July 2	0.9 ± 0.6	28.6 ± 21.4	24.0 ± 22.7	2.1 ± 1.5	1.6 ± 2.4	0.5 ± 0.7
Residues in brood (μg/kg)						
June 17	5.6 ± 0.6	< LOQ	< LOQ	26.1 ± 3.4	1.8 ± 2.6	3.4 ± 4.8
July 2	6.6 ± 2.5	37.0 ± 11.3	25.6 ± 5.3	4.7 ± 0.9	0.7 ± 1.4	< LOQ
Residues in honey (μg/kg)						
May 27	0.8 ± 0.2	< LOQ	< LOQ	< LOQ	28.1 ± 56.1	5.9 ± 4.4
June 3	0.7 ± 0.4	< LOQ	< LOQ	17 ± 7.0	6.3 ± 7.6	1.9 ± 2.5
June 10	2.6 ± 2.8	< LOQ	< LOQ	22.1 ± 14.2	9.1 ± 14.0	1.3 ± 1.5
June 17	0.5 ± 0.7	< LOQ	< LOQ	15.3 ± 9.3	1.0 ± 2.1	1.6 ± 1.9
June 23	1.0 ± 0.3	< LOQ	< LOQ	15.3 ± 10.4	1.1 ± 1.9	0.6 ± 1.3
July 2	4.4 ± 6.0	33.9 ± 37.7	13.3 ± 10.6	16.0 ± 12.2	< LOQ	< LOQ

Recovery studies were performed by spiking the substances used in field trials at a single concentration 0.001 mg/kg of a given matrix. The pesticide residues (*R*_i_) in the samples were recalculated (*R*_rec_) using the results of the recovery study (Rec in %) according to Eq. 1.

1 $$ {R}_{\mathrm{rec}}=100\frac{R_{\mathrm{i}}}{\mathrm{Rec}} $$

% LD_50_ for a single bee (for intoxication by ingestion and by contact) was calculated using Eq. 2.

2 $$ \%{\mathrm{LD}}_{50}={{R_{\mathrm{rec}}}_{\Big( single\  bee}}_{\Big)}\frac{100}{{\mathrm{LD}}_{50}} $$

where *R*_rec (single bee)_ represent residues for a single bee, considering the body weight (b.w.) of a single individual of 0.11 g. Values of LD_50_ for intoxication by ingestion and by contact were taken from literature data (Tomlin 2000; Stoner and Eitzer 2016).

The residue level of a given substance (*R*_rec_), found in samples of honey collected on a given sampling date, was divided by its respective maximum residue level (MRL) (EU Pesticides Database), and a mean percentage of the MRL was calculated using Eq. 3:

3 $$ \%\mathrm{MRL}=100{\sum}_{i=1}^n\frac{R_{\mathrm{rec}}}{\mathrm{MRL}} $$

where *R*_i_ and MRL correspond to the residue level of a given substance in one of the four samples and to the current legally accepted MRL in Poland, respectively. The values of %MRL for all substances (so-called multiple residues) found in each of the four samples were summed, and the total mean percentage of respective MRL values was estimated.

Using the residue level of a given substance (*R*_i_) and assuming a b.w. of 76 kg and a daily consumption (C) of honey by an adult Polish consumer of 0.00157 kg (GUS 2016b, c), long-term dietary intake along with honey was calculated and expressed as %ADI (Acceptable Daily Intake) (Zhu et al. 2015), and then the mean percentages of respective ADI values for each of the four samples collected on sampling days were calculated. Similarly, assuming an additive impact of various pesticides on the human body, the total long-term daily intakes (as %ADI) of all substances were calculated according to Eq. 4:

4 $$ \%\mathrm{ADI}=100\frac{c}{\mathrm{b}.\mathrm{w}.}{\sum}_{i=1}^n\frac{R_{\mathrm{rec}}}{\mathrm{ADI}} $$

Finally, based on the calculated long-term daily intake of a given substance (*R*_i_) with honey expressed as %ADI and daily honey consumption (*C* = 0.00157 kg) by adult Polish consumers, the safe consumption level of the honey (*C*_safe_ in kilograms) was easily calculated using Eq. 5:

5 $$ {C}_{\mathrm{safe}}=100\frac{C}{\%\mathrm{ADI}} $$

## Results

In general, pesticide residue recoveries should be 70–120% of the substance introduced into the sample, and the repeatability should be less than, or equal to, 20% (Document SANTE 2015). In our study, satisfactory values of both parameters were obtained for seven AIs of PPPs in seven sample types. However, for cyprodinil, the recovery in worker bees and honey samples exceeded 120% (respectively 172.1 and 133.4%). Such large differences were the reason for recalculating all the residue values (Eq. 1) to introduce the recovery rate to the results.

### Experiment 1: exposure of a bee colony—red raspberry plantation (Laszka variety)

#### Residues of used pesticides on and in flowers

In flowers of the red raspberry (Laszka variety), surface residues of cypermethrin were found at trace levels of 0.003 μg/flower, but the compound occurred only in samples collected on June 10, i.e. 7 days after application of the insecticide Cyperkil Super 250 EC. Residues of trifloxystrobin (0.040 and 0.011 μg/flower) were found in samples collected on June 3 and June 10, i.e. 1 and 8 days after application of a fungicide Zato 50 WG (Table 3).

The residues of three compounds in raspberry flowers were found: chlorpyrifos (up to 0.012 μg/flower in samples collected on June 3), which was applied to soil as a Dursban 480 EC formulation two times on April 7 and May 15; cyprodinil (up to 0.022 μg/flower in samples collected on June 17), AI in Switch 62.5 WG applied on June 11; and difenoconazole (samples collected on May 27 and June 3, up to 0.023 μg/flower), belonging to triazoles, which was applied as Score 250 EC formulation on May 14 (Table 3).

#### Residues of pesticides on and in leaves

On the leaf surface, only trace residues (0.001 μg/cm^2^ of leaves) of cypermethrin and cyprodinil, and slightly higher residues of trifloxystrobin (0.010 μg/cm^2^ of leaves), were detected (June 10 and 17, June 23 and June 3, respectively) (Table 3).

Incurred residues included chlorpyrifos in samples collected on May 27 and June 3 (on both sampling days, at a mean level of 0.004 μg/cm^2^ of leaves), cyprodinil in samples collected on June 17 and June 23 (up to 0.033 μg/cm^2^ of leaves), and trifloxystrobin at decreasing levels, from 0.042 μg/cm^2^ of leaves (on June 3, the day after treatment) to 0.002 μg/cm^2^ of leaves (on June 23) (Table 3).

#### Residues of pesticides in honeybee samples

Significant residues of chlorpyrifos (up to 7.3 μg/kg of bees in samples from June 17), cypermethrin (only in samples collected on June 10; 2.3 μg/kg of bees), and trifloxystrobin (up to 4.8 μg/kg of bees in samples from June 3) were found in bee samples (Table 3). Using LD_50_ values for bees according to Tomlin (2000) (chlorpyrifos, cypermethrin) and Stoner and Eitzer (2016) (trifloxystrobin), it was calculated (Eq. 2) that the residues of chlorpyrifos in bee samples constituted 1.15% of LD_50_ (contact action) and 0.22% of LD_50_ in the case of substances with gastric activity, while for cypermethrin, the values were 1.27% of LD_50_ and 0.72% of LD_50_, respectively, and for trifloxystrobin were < 0.01% (in this case, Stoner and Eitzer (2016) give LD_50_ for substances with contact action).

#### Residues of substances in brood and honey

In the brood, only chlorpyrifos residues were found in samples collected on June 10 and 17 and amounted to 0.4 and 1.6 μg/kg of brood, respectively, while in honey, apart from chlorpyrifos residues at levels ranging from 2.1 to 4.7 μg/kg of honey (from June 10 to June 23), trifloxystrobin (up to 4.1 μg/kg of honey in samples collected on June 23) was also found (Table 3).

### Experiment 2: exposure of a bee colony—red raspberry plantation (Seedling variety)

#### Residues of pesticides on and in flowers

Among compounds found on flowers (surface residues), the highest residues were determined for boscalid and pyraclostrobin (0.063 and 0.013 μg/flower, respectively), in samples collected on July 2, i.e. 3 days after application of Signum 33 WG, and 10 days after application of Bellis 38 WG, and cypermethrin (residues in samples collected on June 3, i.e. 5 days after Cyperkil Super application amounted to 0.028 μg/flower only, and steadily decreased to the level of LOQ at the end of the experiment). Trace residues of difenoconazole (in samples collected on May 27, i.e. 18 days after Score 250 SC application), and in samples collected on June 10, chlorpyrifos (applied as Dursban 480 EC to soil on May 14) were also found on flowers (both up to 0.002 μg/flower) (Table 4).

Incurred residues of boscalid and difenoconazole were also at the highest levels (both up to 0.018 μg/flower on July 2 and May 27, respectively). Likewise, chlorpyrifos was found in samples (to 0.013 μg/flower in samples collected on May 27), as were pyraclostrobin (0.005 μg/flower in samples collected on July 2) and azoxystrobin (to 0.010 μg/flower in samples collected on May 27, in 20 days after the application of Amistar 250 SC) (Table 4).

#### Residues of pesticides on and in leaves

Among compounds found on leaves (surface residues), the highest residues were determined for boscalid and cypermethrin at 0.057 and 0.055 μg/cm^2^ of leaves on July 2 and June 3, respectively. No trace residues of chlorpyrifos and azoxystrobin at levels exceeding LOQ were found, while difenoconazole residues were determined only in samples collected on May 27 (0.003 μg/cm^2^ of leaves), and pyraclostrobin in samples collected on July 2 (0.025 μg/cm^2^) (Table 4).

In the same leaf samples, incurred boscalid and pyraclostrobin residues (0.033 and 0.019 μg/cm^2^ of leaves, respectively) on July 2 were found. Chlorpyrifos residues in samples collected on May 27 amounted to 0.006 μg/cm^2^ of leaves, and decreased steadily with time to a level < LOQ. Trace residues of difenoconazole were also found in leaves in samples collected on May 27 and June 3 (up to 0.006 μg/cm^2^ of leaves), and azoxystrobin in samples collected on May 27 (0.003 μg/cm^2^ of leaves) (Table 4).

#### Residues of pesticides in bee bodies

Residues of all studied compounds were found in bees foraging on the raspberry plantation (Seedling variety). In samples collected on July 2, boscalid and pyraclostrobin residues were at the highest levels, amounting to 28.6 and 24.0 μg/kg of bees, respectively. Five days after treatment, cypermethrin residues were also at a similar level (22.9 μg/kg of bees in samples collected on June 3) and then steadily decreased, reaching a level of 2.1 μg/kg of bees (Table 4).

Chlorpyrifos residues decreased steadily from 3.9 (on May 27) to 0.9 μg/kg of bees (on July 02), while difenoconazole and azoxystrobin residues, after a respective initial increase to 12.2 and 6.5 μg/kg of bees (on June 3), decreased successively, and on the last sampling date amounted to 1.6 and 0.5 μg/kg bees, respectively (Table 4). Based on the data presented by Tomlin (2000) (for chlorpyrifos and cypermethrin) and Stoner and Eitzer (2016) (for boscalid, pyraclostrobin, difenoconazole and azoxystrobin), according to the calculation using Eq. 2, the maximum residues found in bee samples were 0.61% of LD_50_ for chlorpyrifos (for contact action) and 0.12% of LD_50_ (for gastric action), while for boscalid and difenoconazole for both contact and gastric action, this value was < 0.01% of LD_50_, as in the case of pyraclostrobin and azoxystrobin; the authors here give only the contact dose of LD_50_, with 12.5% of LD_50_ (for contact action) and 7.2% of LD_50_ (for gastric action) in the case of cypermethrin.

#### Residues of the studied substances in brood and honey

On two dates, June 17 and July 2, only the brood was collected from beehives placed in the plantation. All determined AIs were found in the brood, and their levels were similar to those observed in worker bees. In the examined samples, the residues of boscalid, cypermethrin, and pyraclostrobin were highest (37.0, 26.1, and 25.6 μg/kg of brood, respectively) (Table 4).

All studied substances were also found in honey, including chlorpyrifos in samples from each sampling date (up to 4.4 μg/kg of honey in samples collected on July 2). Cypermethrin, difenoconazole, and azoxystrobin were found in samples collected on five dates (up to 22.1, 28.1, and 5.9 μg/kg of honey, respectively), and boscalid and pyraclostrobin were determined in samples collected on the last sampling date (33.9 and 13.3 μg/kg of honey, respectively) (Table 4).

#### Safety of honey consumption

The consumption safety of the obtained honey in terms of formal and legal requirements (Table 5) was evaluated based on the PPP AI residue levels found in honey samples (Tables 3 and 4) and by comparing the obtained results to standards establishing the MRLs for these substances and their ADI (EU Pesticides Database), as well as considering the average honey consumption by an average Polish person.

**Table 5:** Formal and legal evaluation of residue levels of used pesticides found in honey; MRL for all substances: 50 μg/kg

Experiment number		Chlorpyrifos	Boscalid	Pyraclostrobin	Cypermethrin	Difenoconazole	Azoxystrobin	Cyprodinil	Trifloxystrobin
Experiment 1	Residue	4.7	< 0.01	< 0.01	< 0.01	< 0.01	< 0.01	< 0.01	4.1
	%MRL	9.4	< 0.2	< 0.2	< 0.2	< 0.2	< 0.2	< 0.2	8.2
	%ADI	0.01	< 0.01	< 0.01	< 0.01	< 0.01	< 0.01	< 0.01	< 0.01
	*C*_safe_ (kg)	16.2	≫ 16.2	≫ 16.2	≫ 16.2	≫ 16.2	≫ 16.2	≫ 16.2	1853.7
Experiment 2	Residue	4.4	33.9	13.3	22.1	28.1	5.9	< 0.01	< 0.01
	%MRL	8.8	67.8	26.6	44.2	56.2	11.8	< 0.2	< 0.2
	%ADI	0.01	< 0.01	< 0.01	< 0.01	< 0.01	< 0.01	< 0.01	< 0.01
	*C*_safe_ (kg)	17.3	89.7	171.4	171.9	27.0	2576.3	≫ 17.3	≫ 17.3
ADI (μg/kg b.w.)		1.0	40	30	50	10	200	30	100

In samples of honey, only chlorpyrifos and trifloxystrobin residues exceeded 0.2% MRL (9.4 and 8.2% MRL, respectively) in experiment 1, while the residues of the other six substances exceeded 0.2% MRL in experiment 2 (Eq. 3), with boscalid (67.8% MRL) and difenoconazole (56.2% MRL) at the highest level (Table 5).

The health risk to an adult consumer was calculated using Eq. 4 only slightly approached value 0.01% ADI, only for chlorpyrifos (0.010% ADI in experiment 1 and 0.009% ADI in experiment 2), and for difenoconazole (0.006% ADI in experiment 2) (Table 5).

A safe honey consumption level was also estimated, as calculated for each substance using Eq. 5. In general, the lowest consumption level was established for honey from experiment 1, at 16.2 kg. In experiment 2, the lowest consumption level was 17.3 kg (Table 5).

## Discussion

The analysis of samples of flowers, leaves, bees, brood, and honey collected during field experiments showed (Tables 3 and 4) a possibility of diversified transport of applied AIs of PPPs from red raspberry plants to beehives, and their residues were found in brood and honey, analogous to our earlier observations of the transfer of plant protection products from apple orchards (Piechowicz et al. 2018b) and rape cultivations (Piechowicz et al. 2018a). Accumulation of pesticide residues in bee hives appears to depend not only on the pesticide level on a plant but also on daily bee activity, which determines the productivity of the colony.

In field experiments, bees at the plantation for the Laszka variety (experiment 1) were less productive than those located at the raspberry plantation for the Seedling variety, and therefore, the amount of honey found in the hive on June 3 was insufficient to perform a full analysis. On other sampling dates, no significant increase in honey stores was observed. Simultaneously, the studied bees were characterised by considerable aggression. Intensified aggression could be caused by lower productivity in raspberries of the Laszka variety, elicited by lower food availability. The relationship between the lack of food in the hive and intensified aggressive behaviour was described by Collins and Rinderer (1985). In samples collected from that plantation, only chlorpyrifos was found in the brood (June 10 and June 17); in honey (on three and two sampling dates, respectively), chlorpyrifos and trifloxystrobin were determined. Chlorpyrifos (in samples from June 10 and June 17), cypermethrin (in samples from June 10), and trifloxystrobin (in samples from June 3 and June 17) were found in bees. These results were consistent with residues of AIs on and in flowers and leaves (Table 3) in which the compounds appeared on May 27 (chlorpyrifos—in flowers and leaves) and on June 3 (trifloxystrobin, a day after application—on flowers, on leaf surfaces, and on leaf inside). A similar phenomenon was observed at the plantation for the Seedling variety (experiment 2, Table 4), where AIs used in the crop (e.g. cypermethrin applied on May 29 or boscalid and pyraclostrobin applied twice on June 23 and June 30) already appeared on the first day of the sampling after treatment in brood, bee, and honey samples.

Some explanation is required for the trace levels of chlorpyrifos residues, both surfaces and incurred, found in leaves and flowers and in bees, brood, and honey (Tables 3 and 4). This insecticide with deep-seated action was applied at the plantation on April 7 and May 15 (experiment 1) and on May 14 (experiment 2) into the soil through the irrigation system. The main insect targeted by this substance was the May bug *Melolontha melolontha*, whose larvae damage the root system of raspberry bushes. As the obtained results of chemical analyses indicate, chlorpyrifos in this case behaved as a compound with systemic action: it was absorbed by the root system and transported with a transpiration stream to the above-ground parts of plants; therefore, its presence in flowers and leaves was not surprising. As a lipophilic compound, it can penetrate into pollen and nectar and be transferred with them by worker bees to honey storage and the brood inside the hive. This is similar to the mesosystemic trifloxystrobin for which residues in leaves were observed only a day after treatment, but for a longer time on flowers, while inside the leaves, they occurred until the last sampling day. Similarly, cyprodinil, 6 days after the treatment, was observed only inside leaves and in flowers, and only once, on June 23 at the level of the LOQ, on the leaf surface (experiment 1). The systemic AIs in the Seedling variety (experiment 2) behaved similarly. The residues of azoxystrobin, which was applied on May 7, and difenoconazole, applied on May 9, inside the leaves and flowers of the Seedling variety remained there for a longer time than on the surface.

Cypermethrin with contact action most likely did not penetrate the nectar, although it was found on bees (Tables 3 and 4) and in brood and honey (Table 4), likely due to direct contact of honeybees with raspberry leaves and flowers. As Johnson (2015) suggested, bees obtain water to drink directly from leaves. The correlation between the presence of pesticides on the raspberry leaves (Laszka variety) and in the hive was observed by us during other studies (Piechowicz et al. 2018c).

In experiment 2, bees were characterised by much higher productivity, and their colonies were stronger. This is likely due to the much higher activity of the worker foragers than those in colonies placed at the plantation for the Laszka variety or the much higher effectiveness of nectar secreting by raspberries of the Seedling variety. In experiment 2 (Table 4), all applied substances were found not only in workers that had direct contact with the plantation, but also in honey and in brood.

In the raspberry plantation for the Laszka variety (experiment 1), six insecticides were used (two with a systemic effect, three with a contact and alimentary effect, and one deep-seated) (Table 1), and in the plantation of the raspberry of Seedling variety (experiment 2), three insecticides (one systemic, one contact, and one deep-seated) were used (Table 2). The active ingredients of those insecticides belonged to groups of organophosphate (chlorpyrifos, dimethoate), macrocyclic lactone (abamectin), pyrethroids (cypermethrin), chloronicotinyl (thiacloprid), neonicotinoide (acetamiprid), and paraffin oil. Excluding the last compounds, whose action is mainly physical, i.e. blocking of the respiratory system (Card of Characteristics the Preparation Treol 770 EC 2016), the remaining used compounds are neurotoxins. Compounds from the organophosphorus group (e.g. chlorpyrifos applied at both plantations and dimethoate applied at the raspberry Laszka plantation) affect insects by phosphorylation of the acetylcholinesterase (AChE) active site, preventing function. This results in accumulation of acetylcholine in the synaptic cleft, which causes continuous stimulation of the nervous system. Chlorpyrifos contains a P=S group, which during a metabolic oxidation reaction, as part of the animal’s defence system, forms a much more toxic P=O structure. Organophosphorus insecticides could lead to a general perturbation in all systems in organisms (Desneux et al. 2007). Abamectin (avermectins) applied at the raspberry Laszka plantation belongs to the class of macrocyclic lactones and is derived from the soil microorganisms *Streptomyces avermitilis* and *Streptomyces hygroscopicus*. It most likely acts on GABA receptors by activation and blocking of the postsynaptic potential (Fritz et al. 1979) and glutamate-gated chloride channels (Clark et al. 1995). In bees, abamectin also affects cytotoxic midgut cells that may cause digestive disorders in the midgut; epithelial tissue is formed during morphological alterations when digestive cells die and is particularly dangerous for the foraging workers (Aljedani 2017). Cypermethrin applied at both plantations (AI of Cyperkill Super 25 EC) belongs to pyrethroids. The basic targets of pyrethroid action are voltage-dependent sodium channels in nervous tissue (Aldridge 1990; Soderlund et al. 2002) in which the inward sodium current is increased, causing long-term membrane depolarization (Wang and Wang 2003). Pyrethroids are also agonists of T type calcium channels in insect muscles (Aldridge 1990) and reduce the activity of the mitochondrial complex I (Gasner et al. 1997). They also disturb protein phosphorylation (Soderlund et al. 2002) and modify the action of proteins that create intracellular connections of the “gap junction” type (Papaefthimiou and Theophilidis 2001). Nicotine derivatives (thiacloprid and acetamiprid) are agonists of nicotinic acetylcholine receptors in the synapse and hence have an impact on survival, including impaired learning and memory, disrupted navigation, and reduced honeybee foraging activity (Belzunces et al. 2012; Blacquière et al. 2012). Acetamiprid significantly impairs olfactory learning in laboratory-based studies (Decourtye et al. 2005; Han et al. 2010).

The results obtained in both plantations indicate that worker honeybees were exposed to almost all the studied PPP AIs used in the plantation, whose most probably, due to the equipment used, we were unable to detect. Compounds found in the brood and honey were mainly systemic, i.e. those that were transported within a plant and penetrated to nectar and pollen. Numerous studies (Glavan and Božič 2013; May et al. 2015; Zhu et al. 2015) indicate that insecticides are dangerous for bees, while fungicides and herbicides are relatively safe. Despite planters often do not follow the withdrawal period for these compounds, and those engaged in the experiment also did not, the residues were small: Laszka plantation—maximum 1.3% of LD_50_ for bees in the case of contact action and 0.2% of LD_50_ for ingestive action, and at the Seedling plantation—12.6% of LD_50_ and 7.2% of LD_50_, respectively. Both values refer to cypermethrin. As the study indicates, in the case of systemic compounds, conjugates between AIs and plant metabolites can give false results, which can prevent the detection of associated AIs using traditional methods, (Kubik et al. 2000). Additionally, Zhu et al. (2014) suggest that bee resistance against PPPs is ten times larger than in the brood. In addition, performing treatments at the diurnal period of foraging coincides with the period of the highest sensitivity of bees to plant protection products (Piechowicz et al. 2012; Piechowicz et al. 2013; Piechowicz et al. 2016). The preparations used in the studied crops, such as Vertigo 018 EC, Bi 58 Nowy (Eng. New), Cyperkill Super 25 EC, and Dursban 480 WG, are not allowed to be applied during the blossoming period. This is problematic for raspberry because the blossoming period largely corresponds to the period of fruiting. Other preparations can only be used beyond the time of the bee activity. Moreover, an increasing number of authors suggest that interactions of formulations with other PPPs considered safe for bees (Glavan and Božič 2013) are more dangerous than insecticides themselves. In this study, it was not possible to determine all the used AIs, but the obtained results indicated a possibility of interactions between the used insecticides and fungicides. These interactions could have occurred in both experiments between the insecticide acetamiprid and fungicides (Iwasa et al. 2004), thiacloprid (here applied in Laszka variety) and fungicides (Schmuck et al. 2003; Iwasa et al. 2004), as well pyrethroids (here: cypermethrin), or difenoconazole (Colin and Belzunces 1992; Belzunces et al. 2012), used in both plantations. Those interactions can pose even greater threats, as among the formulations are nicotine derivatives, which, as indicated by Kessler et al. (2015), increase the treated plants’ attractiveness for bees. Conversely, Delabie et al. (1985) and Rieth and Levin (1988) indicate cypermethrin as a repellent substance for those insects. In bees, pesticides can also cooperate synergistically with other environmental stressors (Doublet et al. 2015; Renzi et al. 2016).

### Safety of honey consumption

Raspberry honey is characterised by a specific, pleasant smell and is included among the best commercially available honey types. This honey is original and relatively rare. Raspberry honeys obtained from hives installed in the vicinity of commodity plantations may contain pesticide contaminations associated with chemical plant protection.

The highest residue levels were determined for chlorpyrifos and trifloxystrobin in honey from the plantation for the Laszka variety (experiment 1) (Table 3) and for pyraclostrobin, difenoconazole, and cypermethrin in honey from the plantation of the Seedling variety (experiment 2) (Table 4). In no case were the observed values near an acceptable value (MRL = 50 μg/kg). Therefore, in general, in terms of the studied compounds, honey collected for analyses of individual sampling dates met current EU formal and legal requirements. It also met toxicological requirements described by the ADI (Table 5).

## Conclusions

In bee samples, residues of five AIs applied at the raspberry plantation of the Laszka variety were found, three compounds were detected in brood, and two in honey. All six AIs of compounds analysed in the Seedling variety were detected in brood and honey samples. This confirms the active transport of plant protection products from crops to hives and also indicates that bees are exposed to plant protection products from the first days of development. The presence of various AIs, hence probably, also other ingredients included in the PPP formulas in bees, brood, and honey indicate the possibility of synergistic interactions that can markedly increase their toxicity.

The small amounts of pesticides in maturing honey indicate that it is safe for consumption by humans (in no case was the MRL exceeded). This also proves that bees foraging in natural conditions, in specific periods of their life, can be exposed to plant protection products without leaving the hive as a result of consumption of honey containing the residues of pesticides harmful for them.
